# Calcium Binding Promotes Prion Protein Fragment 90–231 Conformational Change toward a Membrane Destabilizing and Cytotoxic Structure

**DOI:** 10.1371/journal.pone.0038314

**Published:** 2012-07-11

**Authors:** Sacha Sorrentino, Tonino Bucciarelli, Alessandro Corsaro, Alessio Tosatto, Stefano Thellung, Valentina Villa, M. Eugenia Schininà, Bruno Maras, Roberta Galeno, Luca Scotti, Francesco Creati, Alessandro Marrone, Nazzareno Re, Antonio Aceto, Tullio Florio, Michele Mazzanti

**Affiliations:** 1 Dipartimento di Bioscienze, University of Milan, Milan, Italy; 2 Sezione di Farmacologia, Dipartimento di Medicina Interna and Centro di Eccellenza per la Ricerca Biomedica (CEBR), University of Genova, Genova, Italy; 3 Dipartimento di Scienze Biochimiche, “Sapienza” University of Rome, Rome, Italy; 4 Dipartimento di Scienze Biomediche, Università “G. d’Annunzio” di Chieti-Pescara, Chieti, Italy; 5 Dipartimento di Scienze del Farmaco, Università “G. d’Annunzio” di Chieti-Pescara, Chieti, Italy; 6 Istituto Superiore di Sanità, Roma, Italy; University of Maryland, United States of America

## Abstract

The pathological form of prion protein (PrP^Sc^), as other amyloidogenic proteins, causes a marked increase of membrane permeability. PrP^Sc^ extracted from infected Syrian hamster brains induces a considerable change in membrane ionic conductance, although the contribution of this interaction to the molecular mechanism of neurodegeneration process is still controversial. We previously showed that the human PrP fragment 90–231 (hPrP_90–231_) increases ionic conductance across artificial lipid bilayer, in a calcium-dependent manner, producing an alteration similar to that observed for PrP^Sc^. In the present study we demonstrate that hPrP_90–231_, pre-incubated with 10 mM Ca^++^ and then re-suspended in physiological external solution increases not only membrane conductance but neurotoxicity as well. Furthermore we show the existence of a direct link between these two effects as demonstrated by a highly statistically significant correlation in several experimental conditions. A similar correlation between increased membrane conductance and cell degeneration has been observed assaying hPrP_90–231_ bearing pathogenic mutations (D202N and E200K). We also report that Ca^++^ binding to hPrP_90–231_ induces a conformational change based on an alteration of secondary structure characterized by loss of alpha-helix content causing hydrophobic amino acid exposure and proteinase K resistance. These features, either acquired after controlled thermal denaturation or induced by D202N and E200K mutations were previously identified as responsible for hPrP_90–231_ cytotoxicity. Finally, by *in silico* structural analysis, we propose that Ca^++^ binding to hPrP_90–231_ modifies amino acid orientation, in the same way induced by E200K mutation, thus suggesting a pathway for the structural alterations responsible of PrP neurotoxicity.

## Introduction

Prion diseases, also called transmissible spongiform encephalopathy (TSE), are fatal neurodegenerative disorders of humans and animals characterized by sporadic, inherited and infective (transmissible) aetiology, including, among the human diseases, Creutzfeldt-Jakob disease (CJD), Gerstmann-Straussler-Scheinker disease, fatal familial insomnia and kuru [1], [2]. The epidemic nature of TSE in domestic and wild animals constitutes a serious health problem also for humans. In fact, in past years the appearance of a new variant of CJD, associated to the consumption of bovine spongiform encephalopathy-contaminated beef, created a troubling new scenario in the transmission of prion diseases [3]. TSE are invariably fatal, with death occurring frequently in less than 1 year after the first symptoms appear [4]. From a neuropathology point of view TSE are characterized by cerebral spongiform degeneration, loss of neurons and gliosis, often associated with amyloid deposition [1]. According to the “protein only” hypothesis [5], TSE share a common pathogenic event: the posttranslational misfolding of a membrane-anchored glycoprotein (cellular prion protein, PrP^C^) into a protease-resistant, aggregation-prone isoform (PrP^Sc^). PrP^Sc^ is considered the main, if not the solely, component of the prion, the infectious entity of TSE [1]. PrP^C^-PrP^Sc^ conversion, physiologically prevented by energy barrier, can occur as a spontaneous stochastic event, possibly favored by mutations in the PrP gene (*PRNP*) or acquired by infection with exogenous PrP^Sc^ molecules. The marked resistance to proteolysis of PrP^Sc^, generating fragments of 27–30 KDa (from which the name of PrP27–30), determines its deposition, as partially cleaved protein, in the extracellular space and the formation of amyloid plaques. Neuronal rarefaction and gliosis occur, although not invariantly, in brain areas where a significant PrP^Sc^ deposition is detectable, suggesting that the protein could trigger the inflammatory and apoptotic cascades [6].

PrP fragments, corresponding to the protease-resistant portion of PrP^Sc^, are widely used to study PrP pathogenic refolding and neurotoxicity. They are characterized by a flexible backbone that can undergo to a conformational rearrangement proposed as a model of PrP^C^-PrP^Sc^ conversion [7]–[10]. Although a definitive general agreement has not been obtained yet, it was proposed that these PrP^Sc^-like fragments can transmit the disease after cerebral inoculation in rodents [11]–[14], supporting the “protein only” hypothesis for the pathogenesis of TSE [5].

We developed a model to study the neurotoxicity of PrP^Sc^ using a recombinant peptide encompassing residues 90–231 of human PrP (hPrP90–231), This peptide, in its native form, is a soluble monomer, mainly structured as α-helix, thus corresponding to a model of PrP^C^ [15]. hPrP90–231 can be converted by mild thermal denaturation (1 hour at 53°C) in a β-sheet-rich conformation that renders the peptide insoluble, highly hydrophobic and partially resistant to proteinase K [10], [16]. All these biochemical features that resembles a PrP^Sc^-like structure, allow hPrP90–231 to acquire biological activities in vitro, inducing microglial activation, astrocyte proliferation and apoptotic neuronal death [7], [17]–[20]. Thus, also considering the recent demonstration that different prion conformations comprise the infectious and neurotoxic entities [21], hPrP90–231 may represent a valuable model to study prion neurotoxicity and the potential development of novel therapeutic approaches [16], [22].

Recently, we reported that Syrian hamster PrP27–30 was able to induce a ionic current through a lipid bilayer and that this effect was not mimicked by hPrP90–231 in its native α-helix-rich, monomeric conformation [23]. However, when incubated in high [Ca^++^] (10 mM), hPrP90–231 induced an effect super imposable to that of PrP27–30 [23]. However, neither the current/voltage relationship nor the relative increases of conductance in high external calcium concentration suggests that the divalent ion directly contributes to membrane permeability [23]. Thus, we hypothesized that the binding of Ca^++^ to the peptide may induce a conformational change in hPrP90–231 allowing a structural conformation similar to the Syrian hamster prion. Numerous studies have implicated alterations in Ca^++^ homeostasis in the neuronal dysfunction in several neurodegenerative diseases, including Alzheimer disease (AD) [24], [25] and TSE [26], [27]. Importantly, in AD, alterations of Ca^++^ signalling were bidirectionally related to the activation of the amyloidogenic pathway 24,28. As far as TSE is concerned, most studies analyzed the effects of PrP^C^ and PrP^Sc^ as regulator (or deregulator) of neuronal Ca^++^ homeostasis (for review see [27]), but a possible role of altered [Ca^++^] in PrP^Sc^-mediated neurotoxicity is still underscored. Age-related deficits in neuronal [Ca^++^] regulatory systems are well characterized, involving higher intracellular [Ca^++^], enhanced Ca^++^ influx through voltage-sensitive calcium channels and reduced capacity of mitochondria to buffer Ca^++^ excess [29]. Recently, it was proposed that Ca^++^ deregulation occurring during the aging process may facilitate the formation of pathogenic Aβ peptides, which in turn further alter neuronal Ca^++^ homeostasis, generating a vicious circle [30] that could occur also during TSE.

The principal aim of this study has been to highlight both the structural features in PrP^sc^ oligomers that push them in the toxic amyloidogenic cascade and the relationship between the acquirement of the toxic conformation with changes in the local environment. For this purpose we used the recombinant hPrP90–231 fragment, taking advantage on its conformational dependent toxicity, as detailed above [7]. We evaluated the role of high [Ca^++^], as determinant of the generation of PrP toxic molecules, analyzing the structural features of hPrP90–231 conformer generated in these experimental conditions that are able to induce the same biological effects of Syrian hamster purified PrP^Sc^. We demonstrate that Ca^++^ binding to hPrP90–231 induces a conformational change in the peptide resembling that previously characterized as the neurotoxic conformer [7], [10]. Importantly, in this PrP^Sc^-like conformation, hPrP90–231 caused cytotoxic and membrane destabilizing effects identical to those induced by Syrian hamster PrP27–30, fully validating the proposed model. Finally, we provide a molecular model of the structural alterations induced by in the PrP molecule after Ca^++^ binding, in comparison with those induced by the disease-related pathogenic mutation E200K.

## Results

### Effect of Calcium on Ionic Currents Elicited by PrP Forms

**Figure 1:**
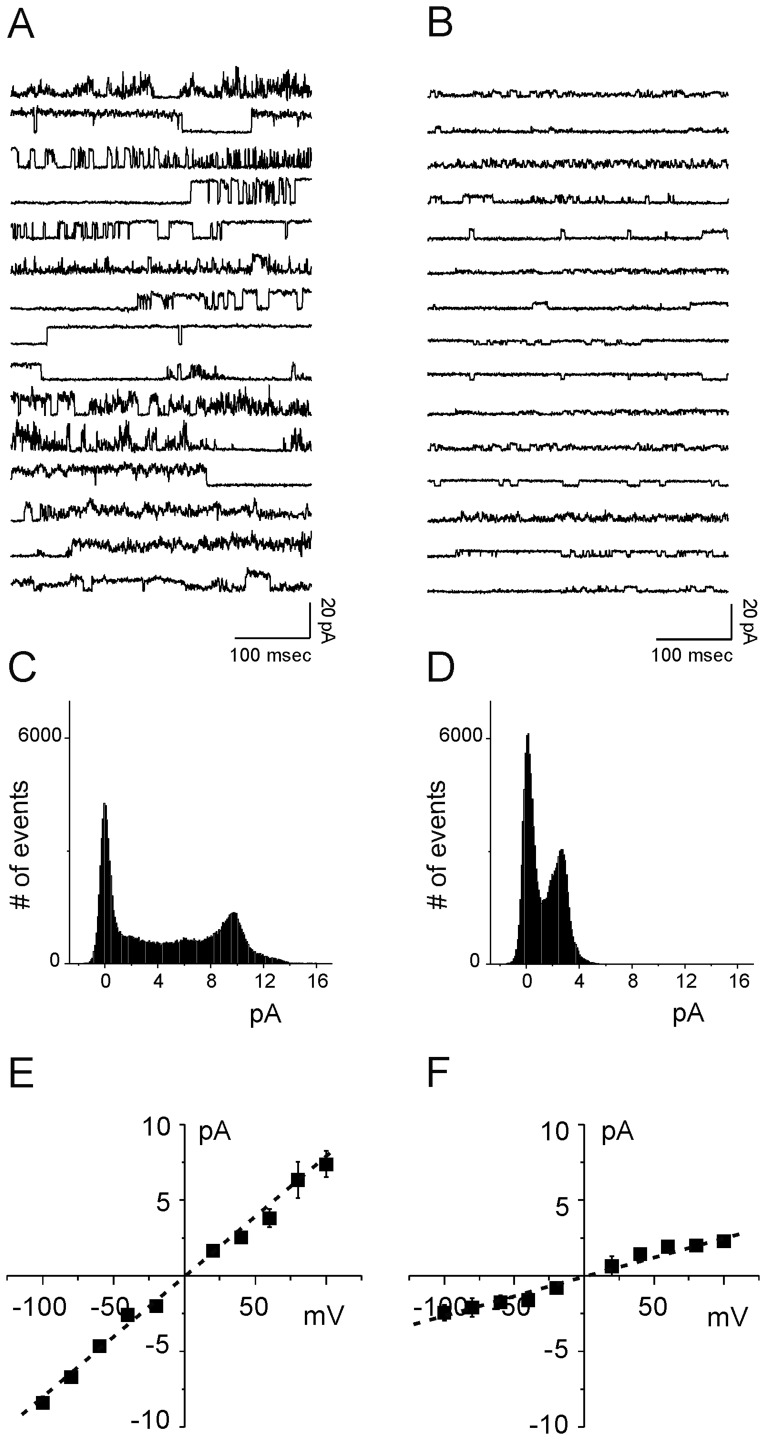
Comparison between PrP27–30 protein and hPrP90–231 peptide in Tip Dip electrophysiological experiments. Fifteen consecutive current recordings obtained at 80 mV membrane potential are shown for infected- brain-extracted protein (panel A) and recombinant PrP peptide (panel B). Panels C and D depict corresponding current amplitude histograms. Histograms were built at each test potential and used to calculate the ionic pathway conductance in the current/voltages relationship showed in the bottom of the figure (panels E and F). From the linear regression of the experimental points (dotted lines) we calculate a conductance of 72±0.23 to 24±0.7 pS for PrP27–30 protein and hPrP90–231 peptide respectively (n = 5).

The Tip Dip technique allows single-channel recordings from artificial lipid bilayer by drastically reducing the surface area available for proteins to insert and, consequently, the background electrical noise. Figure 1 highlight the different ionic permeability induced in artificial membrane of PrP27–30 (left) compared to the recombinant hPrP90–231 (right) in agreement with previous data [23]. We also confirmed that hPrP90–231 conductance increases in elevated calcium ion concentration (10 mM) without apparently increasing the divalent ion flow through the membrane [23].

**Figure 2:**
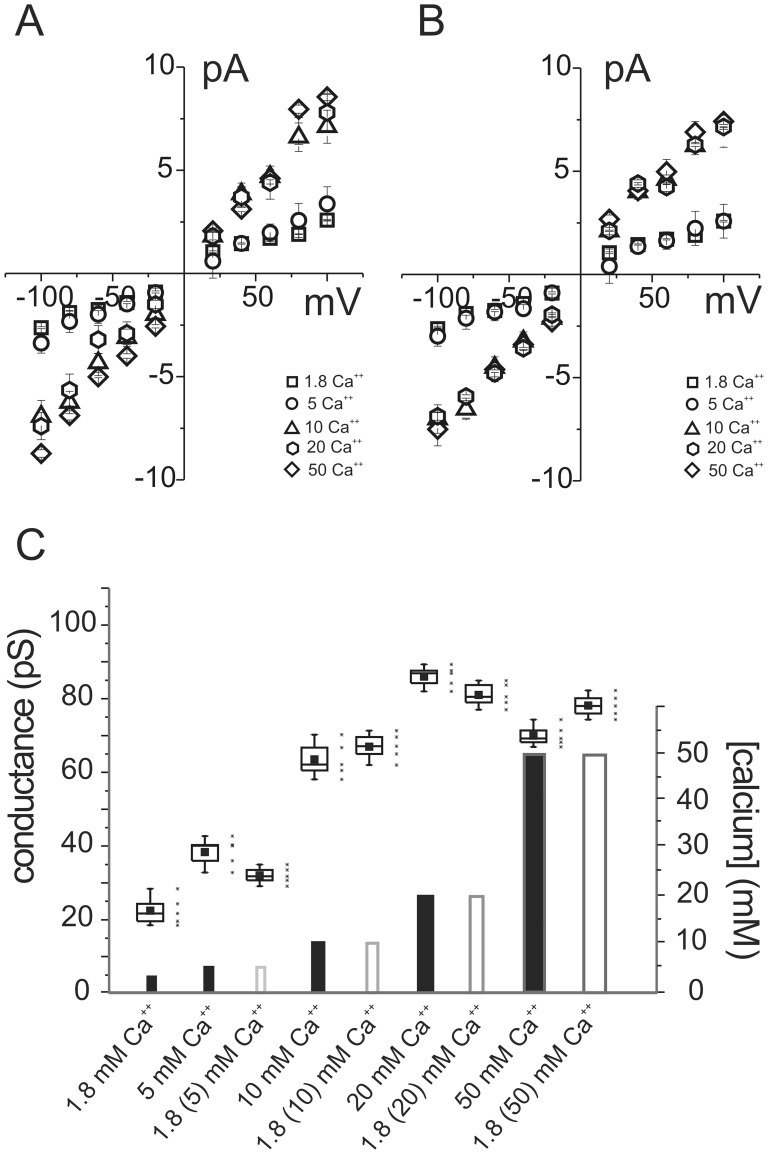
Calcium concentration modulates the ionic conductance induced by hPrP90–231 recombinant peptide. A: current/voltage relationship obtained from Tip Dip experiment using the peptide in 5 different calcium concentrations. B: the current/voltage relationships were obtained from experiments performed in 1.8 mM calcium concentration but the peptide was prior incubated for 1 hour in 4 different calcium concentrations. C: the histogram shows the conductance values versus calcium concentrations either present in the external solution during the experiment or only used during hPrP90–231 incubation. The conductance in the two different conditions show similar values and reaches its maximum at 10 mM calcium. Black and white bars visualize the calcium increase (n = 5).

To separate the effect due to the transmembrane Ca^++^ ionic flow from any other possible interaction of divalent ions and PrP peptide or the influence of high [Ca^++^] on the lipid bilayer, we here recorded single channel events induced by the recombinant peptide in Tip Dip experiments using two procedures. In the first one, a classical dose/response curve was obtained changing [Ca^++^] in the recording pipette. The second procedure consisted in one hour pre-incubation of the PrP peptide in the different Ca^++^ solutions. After this period hPrP90–231 was resuspended (dilution 1∶500) in the control *trans* solution (1.8 mM CaCl_2_) just before performing the electrophysiology measurements. The results in Figure 2 show that the effect of the different [Ca^++^] on membrane conductance is similar either using the divalent ion directly in contact with the bilayer or after one hour incubation. The current/voltage relationships (i/Vs) on the top of Figure 2 depict in both cases two distinct groups of data: current values obtained with the peptide exposed to 1.8 and 5 mM Ca^++^ and a second set of data concerning 10, 20 and 50 mM [Ca^++^]. The histograms in the bottom of Figure 2 highlight the difference between calcium concentration growth (vertical bars) and the increase of single channel conductance (chart boxes). At high [Ca^++^] (10, 20 and 50 mM) conductance values remain constant for both the experimental procedures. Clearly, this cannot be ascribed to a saturation of Ca^++^ permeability since current data obtained using pre-incubated hPrP90–231 (white bars) were recorded in physiological [Ca^++^].

### Effect of Calcium on in Vitro Cell Toxicity of PrP Forms

**Figure 3:**
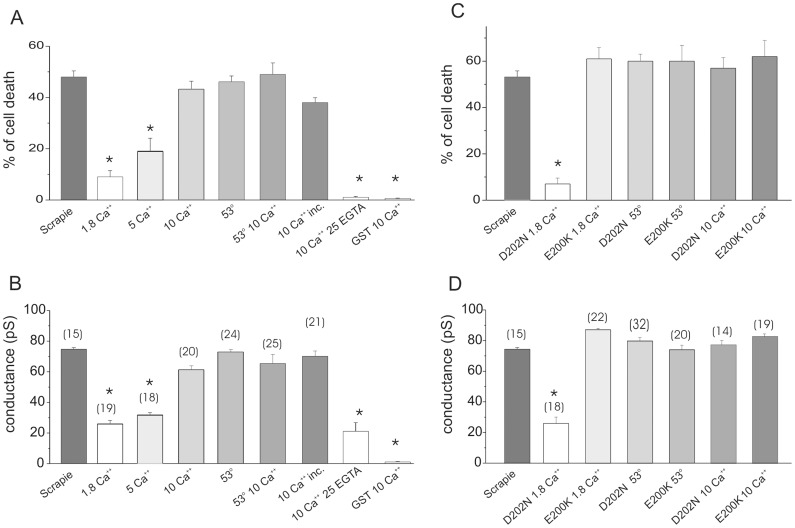
Toxicity versus membrane ionic conductance of the hPrP90–231 peptide. Histograms in panel A and B compare neuroblastoma SH-SY5Y cell death percentage and Tip Dip membrane ionic conductance of PrP27–30 protein (scrapie) with hPrP90–231 peptide in different experimental condition reported below each histogram. In panel C and D the same parallel study was done using two different hPrP90–231 mutants: D202N and E200K. Cell death was measured using the MTT assay after 48 hours of treatment. * = p<0.01 vs. scrapie-induced effects.

Data showed in Figure 2 suggest that membrane conductance increasing following the Ca^++^ treatment is mediated by changes in hPrP90–231 structural features rather than in the lipid bilayer permeability properties. We previously characterized the conformational changes of hPrP29–231 causing gain of toxicity: hPrP90–231 can acquire toxic properties by mild thermal denaturation (53°C, 1 hour) [7], [22], allowing hPrP90–231 to be highly internalized into SH-SY5Y in insoluble aggregates that caused lysosomal dysfunction and cell apoptosis [18]. In Figure 3 panel A the toxic properties acquired by hPrP90–231 after the thermal denaturation were compared with those elicited following the incubation with increasing calcium concentrations. The results showed that the pre-incubation of hPrP90–231 with 1.8 mM Ca^++^ was peculiarly devoid of any effect on SH-SY5Y cell viability while 5 mM Ca^++^ resulted in moderate cell death. Indeed, preincubation with 10 mM Ca^++^ did induce a cell toxicity comparable to the thermal denatured peptide, after 2 days of treatment. These data are all the more meaningful if we consider that the culture medium itself contains high [Ca^++^]. Interestingly, the level of in vitro cytotoxicity induced by thermally denatured and calcium treated hPrP90–231 is similar with that elicited by brain extracted Syrian hamster PrP27–30 (Fig. 3A).

In the histograms of Figure 3B we compare the membrane conductance obtained using the manipulated hPrP90–231 peptide in Tip Dip experiments, using the same treatment reported in the cytotoxicity experiments depicted in Figure 3A. After mild thermal denaturation as well as after pre-incubation in high [Ca^++^] hPrP90–231 drastically increases membrane conductance to a level similar to that induced by PrP27–30. These results suggest that mild thermal partial denaturation and high [Ca^++^] play a similar role on the functional activity of the peptide that seems to be not linked to an increase of calcium ion permeability. We hypothesized that Ca^++^ binding to the peptide is responsible of a rearrangement of its conformation to acquire similar characteristics to those obtained during the thermal denaturation procedure. To probe this hypothesis, we pre-incubated hPrP90–231 in a solution containing 10 mM CaCl_2_. Just before testing the peptide in the Tip Dip apparatus, we diluted (1∶500) the solution to reach 1.8 mM [Ca^++^] (Fig. 3B). Also in these experimental conditions hPrP90–231 greatly affected ionic conductance. Even if thermal denaturation procedure showed a bigger impact on membrane conductance, pre-incubation with 10 mM Ca^++^ caused a substantial increase of ion membrane permeability and the two values that were not statistically different (n = 14). Similarly, applying the same protocol, we demonstrate that hPrP90–231 preincubated with 10 mM Ca^++^and then diluted to 1.8 mM, retains also its toxic effects (Fig. 3A). Finally the effects of incubation with 10 mM Ca^++^ and thermal denaturation were not additive, as far as both cell toxicity and membrane conductance (Fig. 3A and 3B), supporting the hypothesis that the two protocols may induce the same structural alterations. The effects of 10 calcium was prevented by addition of EGTA into the incubation medium. 25 mM of the divalent ion chelator mixed with 10 mM calcium results in a final calcium concentration of 1.6 mM (Clampex, Molecular Device, Novato CA) (Fig. 3A and B). As a control, both in toxicity and lipid bilayer conductance experiments, we use a purified GST protein at the same concentration of hPrP901–231. In three different experimental conditions, 1.8 and 10 mM Ca^++^ as well as after thermal denaturation at 53°C, there was no significant cell toxicity (2% ±5, 4% ±3 and 0% ±9, respectively) neither evident transmembrane ionic flow activity. In Figure 3A and B we report only the results of GST preincubated with 10 mM Ca^++^.

The general idea coming out from our experiments is that a direct link exists between membrane ionic conductance, promoted by the PrP after different treatments, and the ability of the same peptide to be harmful for the cells: larger conductance results in a higher level of toxicity.

Relationship between cytotoxicity and perturbation on the membrane ion conductivity was also tested for single point mutated forms hPrP90–231(E200K), that has been demonstrated [17] to be much more toxic in its native conformation than the *wt* recombinant peptide or the hPrP90–231(D202N) form, bearing a different point mutation (Fig. 3C and D). Partial denaturation procedure or the exposure to high calcium level did not change the E200K mutant powerful effects on cell viability while it causes drastic change in D202N. Conductance values calculated here by Tip Dip technique are 24±2.1 and 80±2.2 pS for D202N and E200K mutants, respectively. Thereby, the membrane conductance change induced by the two mutations proved that while the hPrP90–231(D202N) shows a same conductance value of the w.t. peptide, hPrP90–231(E200K) induces a high level of ion conductivity already in its native conformation paralleling its effects on SHSY5Y cell survival. The exposure to high calcium level did not change the E200K mutant powerful effects on membrane conductance; on the contrary, mimicking the native recombinant, the peptide bearing the D202N mutation increases cytotoxicity and conductance in the presence of both partial denaturation procedure or high calcium exposure (Fig. 3C and D).

### Relationships between PrP27–30- and hPrP90–231 Forms Induced Ionic Current and Cell Toxicity

**Figure 4:**
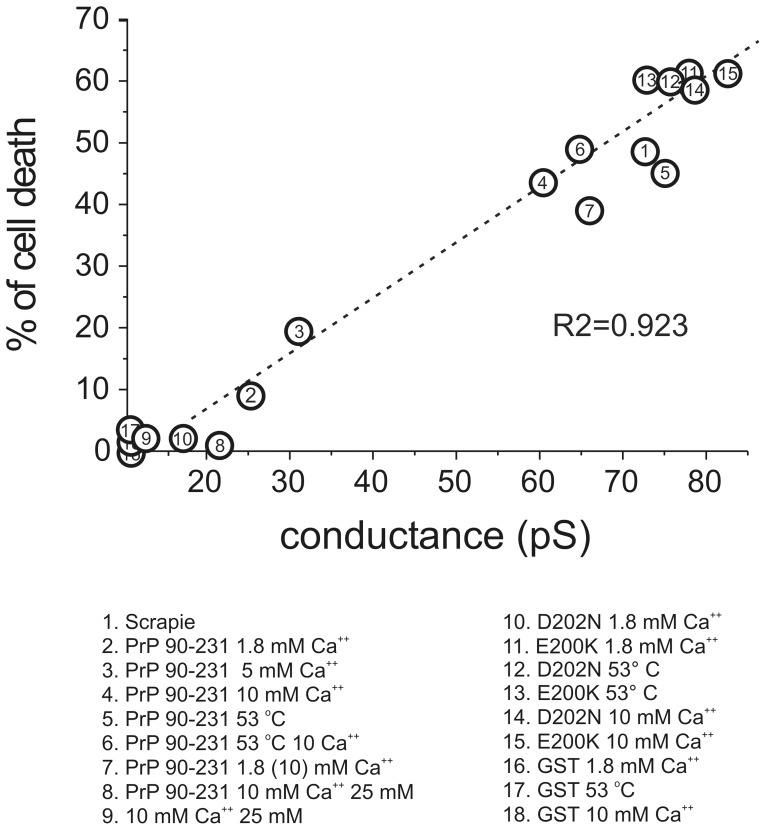
Correlation between toxicity and membrane conductance of hPrP90–231 peptide. All the experimental conditions data reported can be compared with the PrP27–30 protein data (1) representing the native pathological prion protein. Data are plotted as linear regression and statistical evaluation was performed by ANOVA.

From all these experiments cell toxicity and membrane conductance appear to be strictly correlated. To demonstrate this relationship we performed a linear regression analysis comparing the amount of cell death and the ionic conductance of hPrP90–231 *w.t.* and the mutants using the data obtained in all the previous experiments. The plot depicted in Figure 4 shows 18 different data points obtained in parallel electrophysiological and cell survival experiments in several different conditions, including the non pathogenic GST protein. There is a good correlation between the toxicity data and the increase of membrane conductivity operated by the diverse PrP peptides tested in all the different conditions, showing a highly statistical significance (regression coefficient, R^2^: 0.93, P<0.0001, Fig. 4).

**Figure 5:**
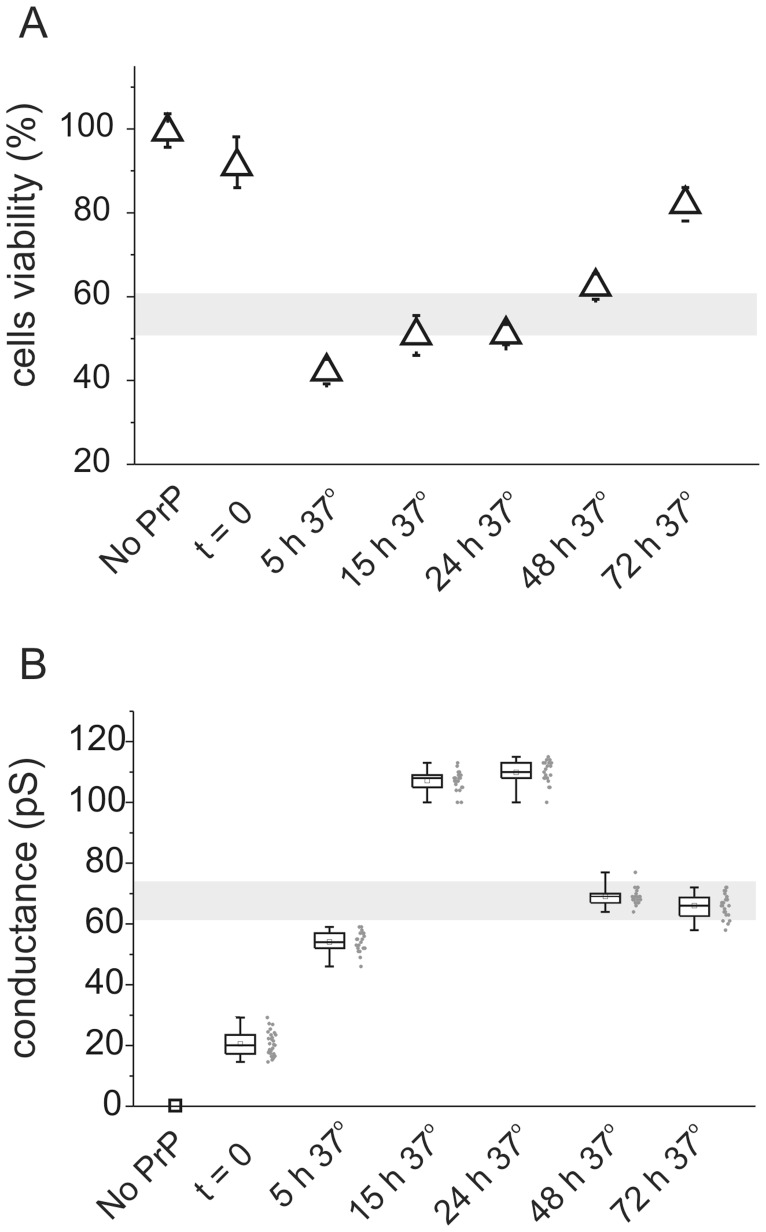
hPrP90–231 peptide incubation time course in physiological condition of cell viability (A) and membrane ionic conductance (B). hPrP90–231 was incubated at 37°C in 1.8 mM Ca^++^, for different times, before being used in cell viability (MTT assay) or Tip Dip experiments. The grey horizontal bar in both plots represents the range of high toxicity (Fig. 5A) and the corresponding range of high conductance (Fig. 5B) obtain treating hPrP90–231 in different conditions (see Fig. 3). The two plots show that between 15 and 24 hours of hPrP90–231 incubation there is a minimum cell viability and a maximum membrane conductance values.

Finally, to demonstrate the relationship between ionic conductance and cell toxicity in experimental conditions in which the structural alterations of hPrP90–231 may spontaneously occur, we evaluated the effects of the peptide after its prolonged incubation at 37°C before being added to the cells (toxicity experiments) or to the lipid bilayer (electrophysiology experiments). In fact, we previously suggested that thermal denaturation at 53°C catalyzes a spontaneous process already occurring at 37°C, reducing the time required for the structural alterations responsible of the gain of toxicity of hPrP90–231 [7]. In line with this hypothesis, we demonstrated that prolonged incubation at 37°C (5 to 24 hrs) favored the acquisition a conformation highly toxic for SH-SY5Y cells in culture (Fig. 5A). Importantly, the same treatment also increased ionic conductance (Fig. 5B), further correlating the two phenomena. For comparison to the two plots were added grey horizontal bands representing the range of high toxicity associated with a large conductance obtained treating hPrP90–231 for a very short time in different conditions (see Fig. 3). Longer pre-incubations at 37°C (5, 15, 24, and 48 hrs) showed a lower response in terms of cell death and higher ionic conductance (Fig. 5A, B). At 72 hours the almost total recovery of cell viability is not followed by a decrease in ionic membrane conductance. This is probably due to the few highly reactive oligomers left during the aggregation process. These results are in line with our previous reports showing that long thermal denaturation treatments favors hPrP90–231 aggregation on preamyloid fibrils [48] that cannot be internalized by the cells and are not cytotoxic [48], [49]. Thus, we can hypothesize that these macroaggregates/prefibrillar structures may less efficiently interact also with the artificial lipid bilayer to induce a reduced ionic conductance.

### Effects of Ca^++^ Binding of hPrP90–231 Structure

**Figure 6:**
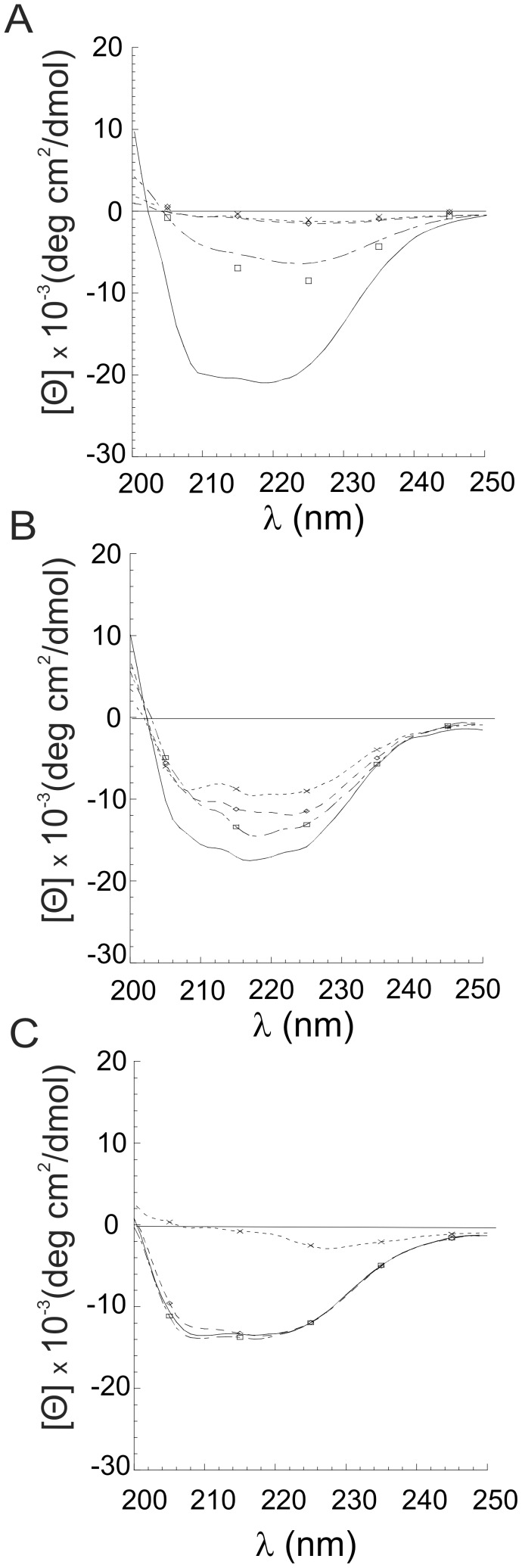
Effect of Ca^++^ on secondary structure of hPrP90–231 *w.t*. (A) and bearing E200K (B) or D202N (C) TSE-related mutations. A. CD analysis of hPrP90–231w.t. in the presence of increasing concentrations of Ca^++^ (mM: 0 = ___; 1.8 = ; 5 = ; 10 = ). **A.** A loss of α-helix structure and increase in β-sheet content was observed in the *w.t.* peptide for increasing Ca^++^ concentrations that was associated, at the highest concentrations to a loss of spectrophotometric signal, likely due to protein aggregation. **B.** CD analysis of hPrP90–231 E200K mutant in the presence of increasing concentrations of Ca^++^. The peptide structure, already enriched in β-sheet content, is not significantly affected by the incubation with the ion. **C.** CD analysis of hPrP90–231 D202N mutant in the presence of increasing concentrations of Ca^++^. Incubation in high Ca^++^ concentration reduce the α-helix content, although showing a lower sensibility than the w.t. protein. At the highest concentration tested a loss of spectrophotometric signal occurs, likely due to protein aggregation.

**Figure 7:**
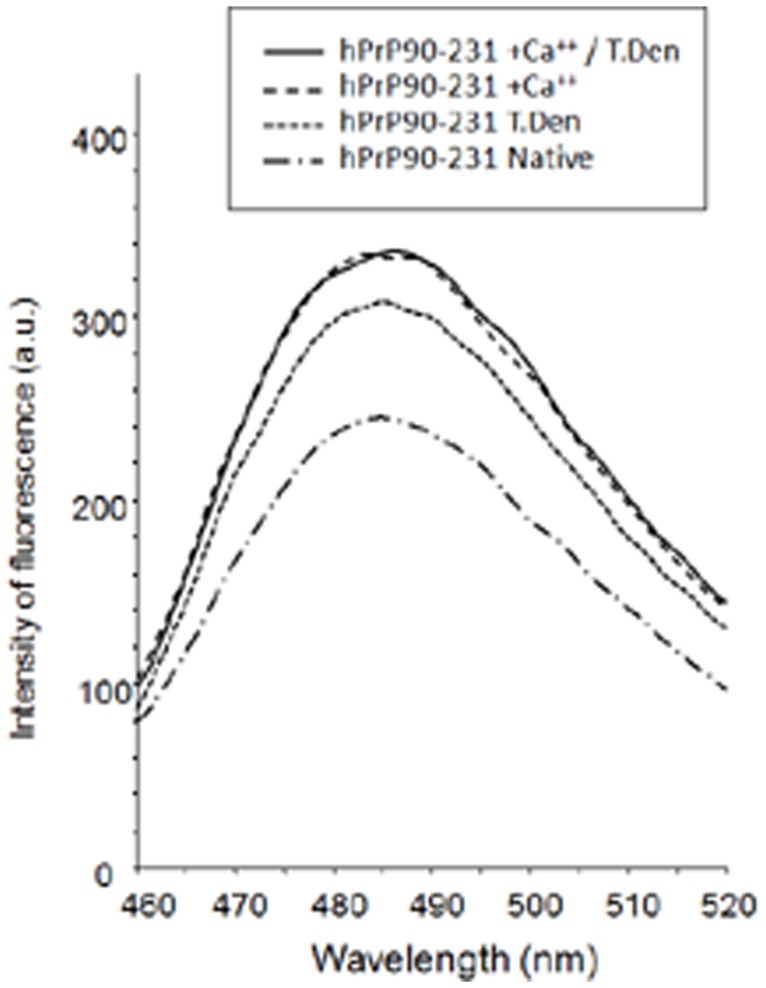
Thioflavin T binding to hPrP90–231 w.t. HPrP90–231 in native, conformation, after mild thermal denaturation (53°C, for 1 hr), after incubation for 1 hr in the presence of 10 mM Ca^++^, or after a combination of both treatments was tested for Th T binding in a fluorimetric assay, as index of β-sheet content. As previously reported, thermal denaturation increased the Th T binding as compared to the native peptide, but this effect was slightly higher after incubation with Ca^++^. No additivity between thermal denaturation and incubation in the presence of 10 mM Ca^++^ was observed.

To directly monitor the effects of Ca^++^ on hPrP90–231 structure, we analyzed several parameters that previously we demonstrated to be determinant for the gain of toxicity of hPrP90231. By CD analysis, we demonstrated that hPrP90–231, which in its native conformation is mainly α-helix structured (Fig. 6A), in the presence of increasing [Ca^++^], loses its structuration. A similar structural change occurred after thermal denaturation at 53°C [7]. Higher [Ca^++^] caused a reduction of the spectroscopic signal likely due to the aggregation of the peptide. By means of ThT binding assay, as index of β-sheet content, we also show that incubation of hPrP90–231 with 10 mM Ca^++^ induced an increase in the fluorescence emission (C-max) slightly higher than that induced by thermal denaturation (Fig. 7), suggesting the likely occurrence of increasing in the β-sheet content as a consequence of incubation in high [Ca^++^] as demonstrated after thermal denaturation. In fact, no additivity on ThT binding was observed combining the two treatments on hPrP90–231 (Fig. 7).

**Figure 8:**
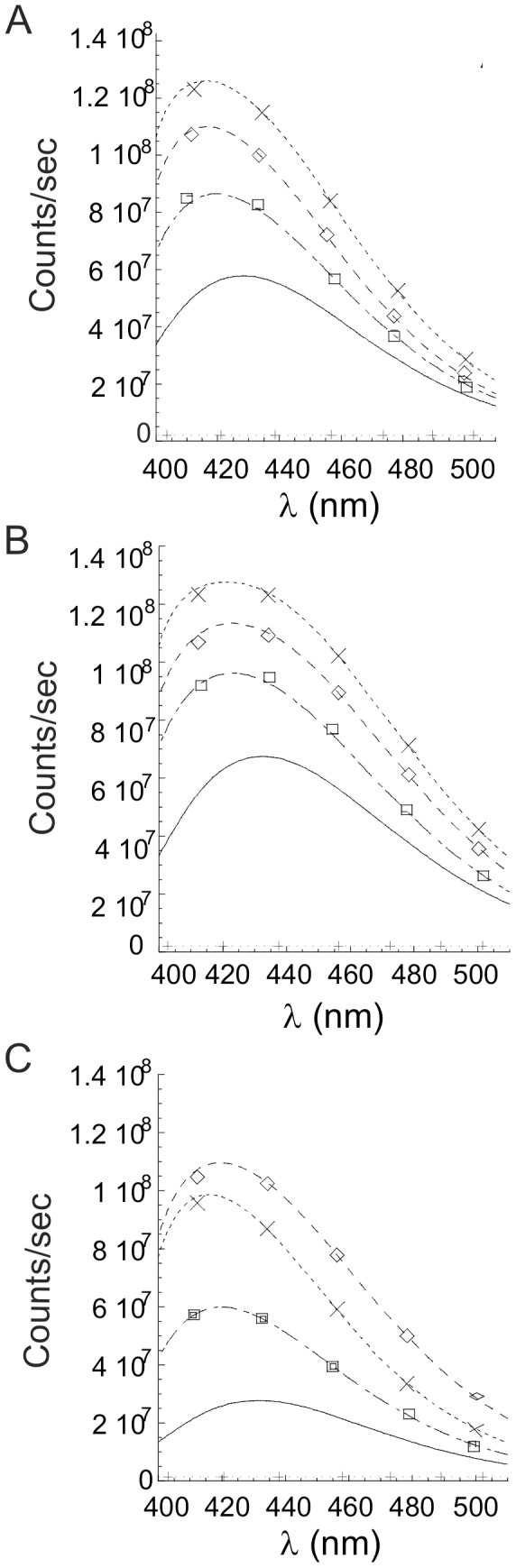
Effect of Ca^++^ on hydrophobic amino acid exposure, measured by TNS binding, of hPrP90–231 *w.t*. (A) and bearing E200K (B) or D202N (C) TSE-related mutations in the presence of increasing concentrations of Ca^++^ (mM: 0 = __; 1.8 = ; 5 = ; 10 = ). As internal control, the direct effect of 10 mM Ca^++^ on TNS is reported (+). **A.** TNS binding assay of hPrP90–231 w.t after incubation with increasing concentrations of Ca^++^ show a great increase in TNS binding, indicating the increased hydrophobicity of the peptide. **B.** TNS binding assay of hPrP90–231 E200K showed a basal higher hydrophobicity than the *w.t.* peptide, although in the presence of increasing concentrations of Ca^++^ TNS binding greatly increased, indicating increased hydrophobicity of the peptide **C.** TNS binding assay of hPrP90–231 D202N in the presence of increasing concentrations of Ca^++^. In these experimental conditions the hydrophobicity of the peptide greatly increased, as indicated by the TNS binding fluorescence although reaching lower levels than observed with the other peptides.

hPrP90–231 N-terminus structural unfolding in the presence of high [Ca^++^] was also monitored by measuring the intrinsic fluorescence of the unique tryptophanylic residue located in the position 99 of the hPrP90–231 sequence. Incubation of the peptide with 1.8, 5 and 10 mM Ca^++^ caused a concentration-dependent increase in fluorescence emission that was comparable to that induced by thermal denaturation (data not shown). Similar results were also obtained evaluating the hydrophobicity of the peptide: as observed after thermal denaturation [7], increasing [Ca^++^] induced a higher hydrophobic amino acid exposure on hPrP90–231 surface as measured by TNS binding (Fig. 8A).

**Figure 9:**
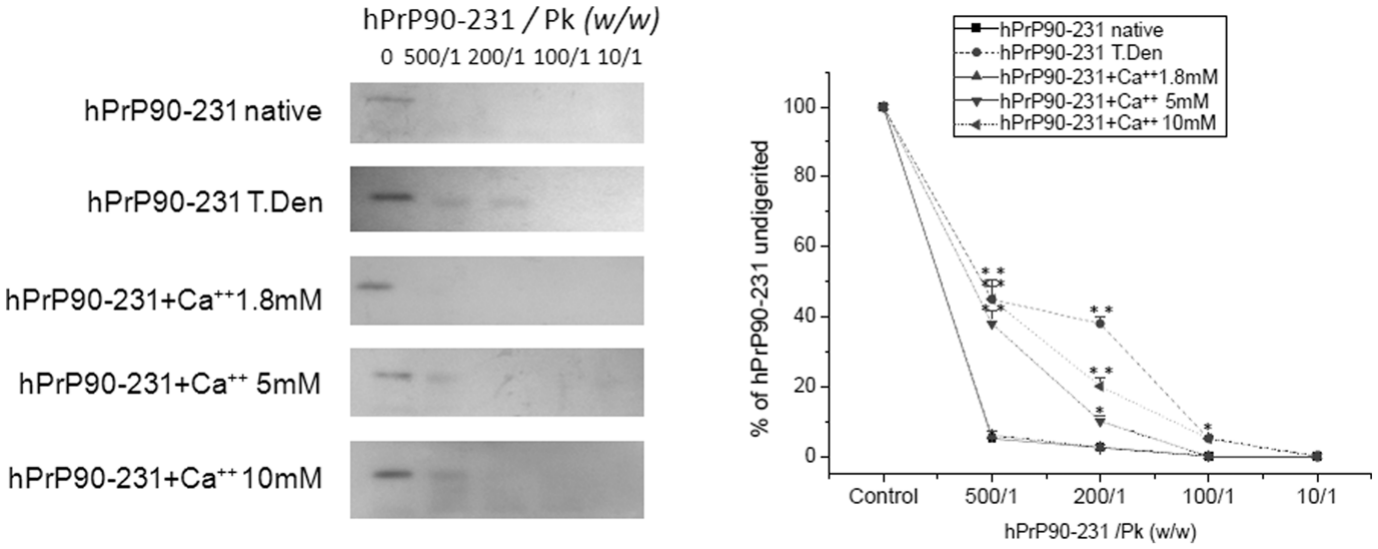
Increased proteinase K (PK) resistance of hPrP90–231 *w.t.* in the presence of increasing Ca^++^ concentrations. hPrP90–231 w.t. was thermally denatured or preincubated with increasing concentration of Ca^++^ before being subjected to PK digestion with different enzyme concentrations. Partial resistance was evaluated in Western blot experiments using the anti-PrP antibody 3F4. Left panel shows representative immunoblots, while the graph on the right depicts the densitometry analysis of three independent experiments. Data are expressed as percentage of the respective recombinant peptide input. * = p<0.05 and ** = p<0.01 *vs*. control values. In native conditions or in the presence of low Ca^++^ concentrations, the peptide showed no resistance to PK digestion. After incubation with 5 or 10 mM Ca^++^ a partial resistance was observed for a peptide/PK ratio of 500∶1. Thermal denaturation induced a higher resistance as compared to Ca^++^ binding, being the immunolabeled peptide band detectable also for a peptide/PK ratio of 200∶1.

Finally, one of the hallmarks of pathogenic PrP is proteinase K resistance, which can be induced in hPrP90–231 by thermal denaturation [7]. Incubation with Ca^++^ (5 and 10 mM) significantly increased the resistance of hPrP90–231 to proteinase K digestion, with the effect induced by the concentration of 10 mM comparable to that caused by incubation at 53°C for 1 hr (Fig. 9). Conversely, hPrP90–231 in native conformation, or after the incubation with low [Ca^++^] (1.8 mM), was completely digested even at the lowest PrP/PK ratio (500/1) (Fig. 9).

Similar results were obtained using hPrP90–231 mutants. E200K mutation that *per se* shows high TNS binding (Fig. 8B) and tryptophan fluorescence (data not shown) showed a moderate further increase in these values, reaching the same values observed in the *w.t.* peptide (Fig. 8A). We previously reported a similar behavior also after thermal denaturation [17]. As far as secondary structure, hPrP90–231 E200K that is natively rich in β-sheet [17], after incubation in high [Ca^++^] no significant changes were observed as far as β-sheet content although a loss of signal was observed, likely due to protein aggregation (Fig. 6B). Again a similar protein behavior was detected in previous studies after thermal denaturation [17].

D202N mutation that mildly affected native hPrP90–231 structure [17], acquired increased hydrophobicity (Fig. 8C) and N-terminal tryptophan exposure (data not shown) after incubation in high [Ca^++^], resembling the structural alterations observed after thermal denaturation [17]. Interestingly, CD analysis showed that α-helix content remained unchanged in the presence of Ca^++^ (Fig. 6C), as was previously described after thermal denaturation [17]. Thus, to conclude this part of the study we propose that the biological effects induced by hPrP90–231 incubation in high [Ca^++^] are due to a structural rearrangement that induce a conformational change closely related to the toxic conformation we previously described after mild thermal denaturation [7], [17], [48].

### Molecular Modeling

Here, the structural and electrostatic changes occurring as a result of the E200K mutation and the binding of the Ca^2+^ ion on the PrP90–231 structure were analysed, in comparison to the *w.t.* protein, by means of molecular mechanics based approaches and electrostatic potential similarity analyses. The computational protocol we used is based on the combination of LLMOD searches and multiple minimizations and allowed to develop reliable models for (i) the wild type PrP90–231 protein (model I) and its E200K mutant (model II), whose NMR structures are already well known [38], [39], and (ii) the binding of the Ca^++^ ion by the Pr90–231 segment of PrP^C^ (model III) that is still unknown and is the main goal of this study. The similarity between the electrostatic potential at the proteins surface was then evaluated for these models employing the Carbó similarity index (see Material and methods). This procedure allowed us to propose a molecular model for the binding of the Ca^++^ ion to PrP^C^ protein and suggest possible structural and electrostatic alterations responsible for the increased neurotoxicity.

### Structural, Charge and Similarity Analyses of w.t. PrP^C^ and the E200K Mutant

**Figure 10:**
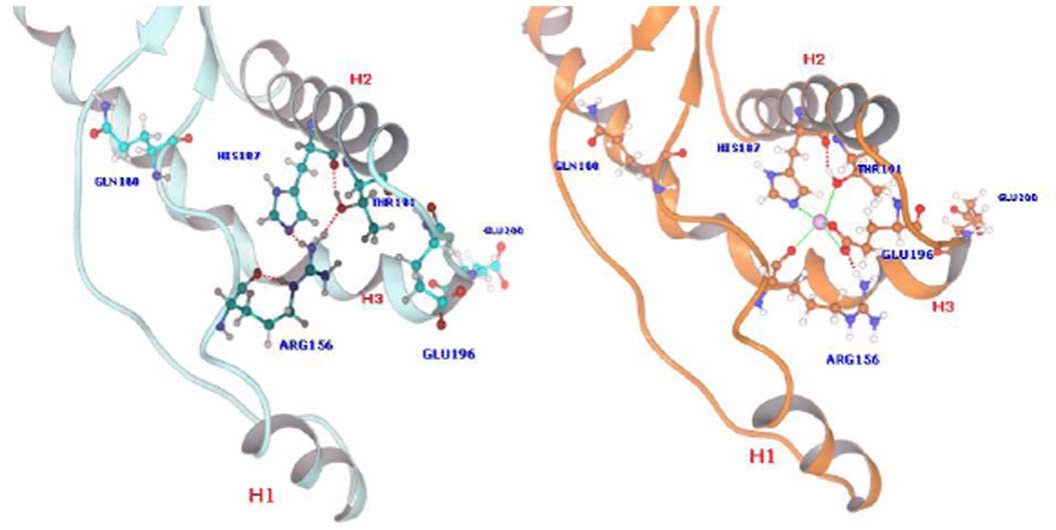
Structure of the proposed Ca^++^ binding site in the wild type PrP90–231 protein. On the left the site with the residues still involved in an H-bond pattern in model I (light cyan); on the right the resulting complex after Arg156 shift and Ca^++^ binding in model III (orange). Hydrogen bonds (dotted red) and coordinative bonds (green) are explicitly indicated.

Preliminarily, we evaluated the predictive performance of models I and II in reproducing the experimental behaviour of the *w.t.* hPrP90–231 protein and its E200K variant. Both proteins include a globular domain, residues 125–228, containing three α-helices comprising the residues 144–154 (H1), 173–194 (H2), and 200–228 (H3) and a short anti-parallel β-sheet comprising the residues 128–131 and 161–164 (Fig. 10, left panel). A comparison of the NMR structures of these two proteins shows, in spite of the Glu200→Lys substitution, an almost identical overall structure, even in the vicinity of residue 200. Indeed, except for the loss of the Glu200-Lys204 salt-bridge interaction within the H3 helix and significant alterations in the highly flexible loop 167–171, only minor differences in interdomain interactions are observed. Particularly interesting is the H-bond pattern, involving the Arg156, His187 and Thr191 residues and connecting the C terminus of H1 and H2 in the *w.t.* protein (Fig. 10, left panel), that is lacking in the E200K variant Fig. 10, right panel). These structural differences are all nicely reproduced by our models I and II thus validating our MM approach.

In spite of the almost identical three-dimensional structure, the Glu200→ Lys substitution has important effects on the charge distribution at the protein surface. Indeed, the replacement of the negatively charged Glu with a positively charged Lys residue results in a dramatic redistribution of the surface charges introducing patches of positive potential around the beginning of helix 3, and has been suggested to facilitate the PrP^C^ to PrP^Sc^ conversion by promoting protein-protein interactions and aggregation [39].

The effects of the mutation on the electrostatic properties of PrP^C^ was then investigated in detail by analyzing the similarity between the molecular electrostatic potential (MEP) near the surface of the *wild type* (model I) and the E200K mutant (model II). Such an analysis was the performed by calculating the Carbó similarity index (σ*_C_*) [47] (see Materials and Methods) between the two MEPs on the points of a grid encompassing the superimposed protein models (I U II). Moreover, because the H3 helix has been suggested to play a key role in the disease-related conformational transformation of PrP^C^ to PrP^sc^ in vivo [50], and is also directly involved in the considered E200K mutation and (see below) in the binding of Ca^2+^ ions, we have perform our similarity analysis with respect to its axis (approximately corresponding to the x-axis in the workspace coordinates of Maestro, see Computational Details). Accordingly, the grid space was partitioned into slices perpendicular to the x axis, and the similarity index was calculated on each slice and the MEP similarity was monitored as a function of their position along the x axis.

**Figure 11:**
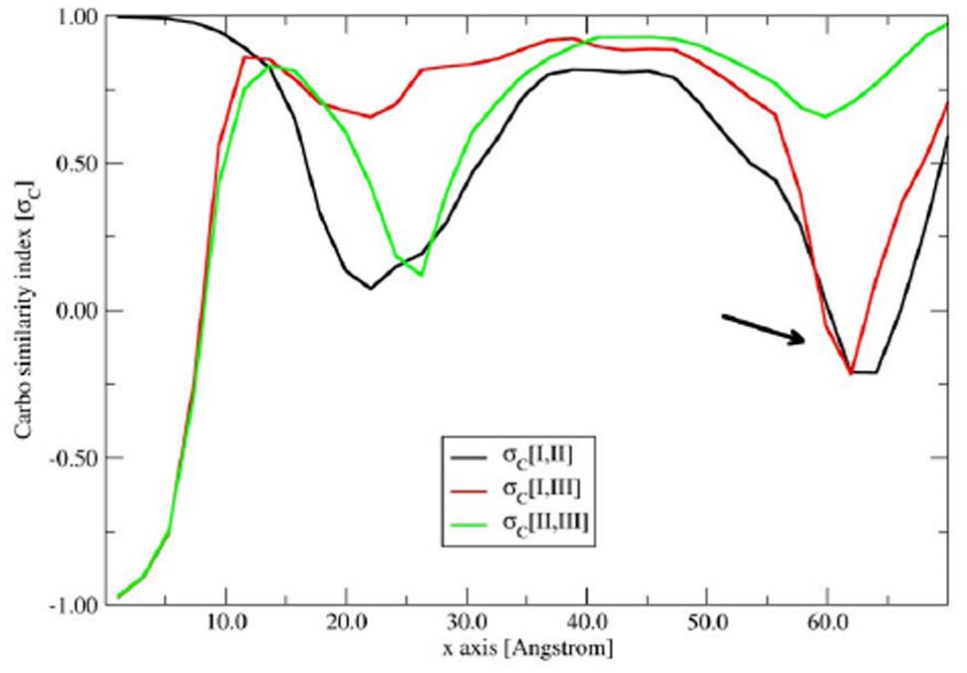
Plot of the Carbó similarity index (σC) for the MEPs of models I-III as a function of the position along the x axis.

The plot of the I/II similarity index, reported in Figure 7, shows two minima at 22–23 Å and at 62–63 Å along x axis, at the beginning and close to the C terminus of H3, respectively (see Figure 11 and S2).

The first minima is due to the completely different orientation of the charged Glu168 and the Tyr169 residues in the highly flexible loop 167–171 which significantly differs in the two protein forms. More importantly, the lowest minimum at 62–63 Å, characterized by a negative value of –0.25 of the Carbó index (indicating a large MEP similarity) encompass the region around the protein surface around H1, the C terminus of H2 and the beginning of H3, and comprising the residue at position 200 (Fig. S2). This region also includes the Arg156, His187 and Thr191 residues involved in the H-bond pattern connecting the C terminus of H1 and H2 in the *w.t.* protein (model I) and switched off in the E200K mutant (model II). The presence of a deep I/II similarity minimum in this region indicates that significant changes in the charge distribution has occurred therein as a consequence of E200K mutation, and is not surprising because of the charge inversion associated to the E200K mutation and the breaking of the H-bond pattern connecting H1 and H2. The graphical inspection of the protein in this region revealed a significant shift of Arg156 as a consequence of E200K mutation: indeed, while in model I the side chain of this residues is directed toward the protein interior, in model II it protrudes outside the protein surface and this shift could contribute to the negative values of I/II similarity index around 62–63 Å.

### Binding of Ca^++^ to the Wild Type PrP^C^

The building of a molecular model for the binding of the Ca^2+^ ions to the PrP^C^ wild type protein (model III) was carried out on the basis of an inspection of the MEP and of the distribution of potentially coordinating side chains around the *w.t.* protein surface, suggesting a plausible metal binding site in the close proximity of His187, Thr191 and Glu196. Although His187 and Thr191 are involved in an H bond pattern with Arg156, that also contributes to keep them in close proximity (Fig. 10, left), this pattern can be easily broken, as shown by the analysis of the E200K mutation above. We thus hypothesized that Arg156 guanidine could shift from its position in the *w.t.* PrP^C^ allowing the Ca^2+^ ion to coordinate the His187 and Thr191 and the nearby Glu196. The structure of the resulting PrP^C^-Ca^2+^ complex (model III) was calculated by using the same computational approach employed for the calculation of models I and II. The Ca^2+^ coordination site resulting by our computational approach involved His187, Thr191, Glu196 (η^2^ coordination) and the backbone carbonyl oxygen of Arg 156 arranged in an distorted square-planar coordination (Fig. 10, right).

The effects of Ca^2+^ coordination on the electrostatic properties of PrP^C^ were evaluated by the analysis of the MEP similarity of model III with models I and II using the same approach described above. The most important feature of the analysis of the I/III MEP similarity as a function of their position along the x axis is the presence of a negative minimum at 62–63 Å, with the same position and similarity values of the minimum observed for the I/II similarity (Fig. 11). This result clearly indicates that, the effect of Ca^2+^ coordination is very similar to that induced by the E200K mutation, leading to a redistribution of the surface charges with patches of positive potential around the beginning of H3.

This conclusion was further supports by the analysis of the of II/III MEP similarity which showed relatively high similarity values (higher than 0.50) at 60–65 Å along the x axis, indicating similar electrostatic properties of models II and model III at the beginning of H3.

## Discussion

PrP misfolding is the pathogenic event responsible of the clinical and pathological features of TSE, including neurotoxicity and transmissibility [51]. To date, the exact mechanisms by which the transition PrP^C^→PrP^Sc^ may occur, as well as the final conformation providing PrP^Sc^ with gain of toxicity have not been completely elucidated. However, in the past years, several studies using recombinant PrP molecules highlighted potential mechanisms by which misfolded PrP may cause cell death and potential molecular determinants of such effects [51]. We developed an experimental model to study PrP neurotoxicity using an amino-terminally truncated PrP isoform (PrP90–231) that can transit from a PrP^C^-like to a PrP^Sc^-like conformation by mild thermal denaturation. In these conditions hPrP90–231 become β-sheet structured, highly hydrophobic, amyloidogenic and neurotoxic. In this study we analyzed different and possibly more physiological mechanisms of hPrP90–231 transition toward the neurotoxic conformation, evaluating the role of Ca^++^ in this process. Moreover, comparing the effects of the recombinant protein with PrP27–30 isolated from infected hamster brain, we identified an altered ion current in synthetic lipid bilayers as marker of the interaction of neurotoxic PrP molecules with cell membranes.

It is a whispered characteristic of amyloid compounds to be able to interact with lipid bilayers [52]–[54]. Not only amyloid compounds are found in close association with cell membrane but also they are responsible to cause a vast variety of electrophysiological transmembrane signals [55]. PrP is not an exception. Several investigations report the ability of PrP to induce changes in ionic conductance both in artificial [56], [57] and cell [58] membranes. Whether PrP is able to form structures similar to conventional ion channels is still debatable. Whether the induction of ion flux is part of the toxicity mechanism responsible for cells death during insurgency of the pathological condition it is even more controversial [59].

In a previous report we demonstrate that PrP^Sc^ purified from infected hamster brain is able to open ionic conductance in which the most probable current level is around 80 pS. On the contrary the recombinant peptide, hPrP90–231, composed by the same amino acid primary sequence shows an average conductance of 20 pS [23]. In the present investigation we confirm these data and correlate the ionic conductance value with the toxicity level obtained in neuroblastoma cell culture. Cell death percentages follow proportionally the measured conductance calculated for the different conditions in which we treated the PrP90–231. Pathological PrP^Sc^ appears to be 5 folds more harmful when compared with recombinant peptide in its native, α-helix structured conformation. The arrangement of the secondary structure of PrP peptides is known to determine their cytotoxicity. Recombinant hPrP90–231 peptide is a versatile model to study the conformation-dependent toxicity [7], [10]. In a previous study it was demonstrated that both mild thermal denaturation and the conformational changes induced by TSE-related point mutations drastically increases cell toxicity [17]. Here, using hPrP90–231 in Tip Dip experiments, we demonstrate that a higher level of cell death correspond to a higher membrane conductance values reaching values comparable to those induced by PrP 27–30. Importantly, similar results were obtained incubating *w.t*. and mutant hPrP90–231 molecules with increasing Ca^++^ concentrations. In these experimental conditions PrP truncated peptides increased ionic conductance and toxicity, with a highly significant direct relationship. Since these experimental conditions determine a rearrangement of the tridimensional structure of the peptides, we can conclude that the key conformational features present in the hamster prion responsible of the membrane interaction are likely reproduced in hPrP90–231.

This is the first evidence that biological and electrophysiological characteristics of brain extracted PrP27–30 can be entirely reproduced by recombinant PrP fragments. Moreover, these results further support the use of hPrP90–231 for identification of the mechanisms of neuronal dysfunction and neurotoxicity in TSE patients and for the identification of potential new pharmacological approaches to be translate in clinical setting [22], [49].

The second relevant observation coming out from this study is that the same neurotoxic conformation we previously described to be induced by mild thermal denaturation can be reproduced in the presence of high [Ca^++^]. In particular, accordingly to our molecular modeling, to induce hPrP90–231 gain of toxicity, Ca^++^ binding to the recombinant peptide might induce hPrP90–231 aggregation that, in turn, is responsible of alterations in the peptide three-dimensional structure, increasing of its hydrophobicity, and, finally inducing a partial resistance to protease K digestion. These observations suggest two additional considerations: 1) hPrP90–231, likely due to the N-terminal truncation, show reduced energy barrier and thus is more prone to adopt a neurotoxic conformation in different environmental conditions (mild thermal denaturation, high divalent ion concentrations); 2) the highly reproducible induction of the same biological effects by hPrP90–231, independently of the means used to induce the conformation change, suggest that this transition may represent the (or one of the) neurotoxic PrP conformation(s) *in vivo*.

In this scenario the role of Ca^++^ on PrP peptide function it is of fundamental importance. Increase of [Ca^++^] from physiological levels up to 10 mM is directly responsible of the conformation changes that allow acquisition of the two main biological features identified in hPrP90–231: activation ionic conductance and increased toxicity. To understand how Ca^++^ binding can induce such a dramatic structural alteration, we performed *in silico* structural analysis comparing the amino acid charge distribution of PrP *w.t.* in the absence or presence of Ca^++^ binding in a specific coordination site, with the model of PrP E200K. In fact, from previous studies we identified two TSE-related PrP mutations (D202N, E200K) that differently affected PrP α-helix 3 stability [35]. Importantly, E200K mutation induces in hPrP90–231 a spontaneous acquisition of the toxic and ionotropic conformation, independently from thermal denaturation [17] or Ca^++^ binding. Thus, we verified were the three-dimensional alteration induced by the E200K mutation could be mimicked by Ca^++^ binding to PrP90–231. Our *in silico* study confirmed this hypothesis showing that Ca^++^ binding can occur in proximity of PrP pavement, in an interdomain region connecting H1, H2 and H3 domains. The analysis of electrostatic similarity in model I, II and III showed also that both Ca^++^-bound *w.t.* protein and the E200K mutant are characterized by a significant similarity in the pavement region. This result indicates that either the mutation or Ca^++^ binding exert similar effects in the charge distribution on protein surface. Importantly, our results indicated that both E200K substitution and Ca^++^ binding may reduce the strength of H1–H2 and H1–H3 interactions by a similar mechanism of electrostatic destabilization affecting the conformational stability of the protein and, thus, facilitating the PrP^C^ →PrP^Sc^ transition.

Consistently with this electrostatic interpretation, it is expected that the binding of other bivalent metal ions, such as Mg^++^, Zn^++^ or Cu^++^ (whose coordination features can be considered similar to Ca^++^) can also induce analogous effects on *w.t.* protein. This observation could be particularly relevant for Cu^++^ considering the known modulator role of this ion on PrP folding and, possibly, function [60]–[63]. The evaluation of Cu^++^ effects on hPrP90–231 structural, biochemical and biological function is currently in progress.

Thus, using several different experimental approaches, we demonstrated that Ca^++^ binding to hPrP90–231 may favor the conversion of hPrP90–231 in a neurotoxic conformer. However, the experimental conditions responsible to convert PrP peptide in the harmful form are rather extreme (maximum effect at [Ca^++^] 10 mM) and it is unlikely to find such a high concentration of the divalent ions or such a high temperature (53°C in the thermal denaturation model) in a living organism. However, two considerations should be taken in account. As shown in Figure 5, the conversion of the peptide in the toxic form can take place even in “physiological” conditions, such as prolonged incubation at 1.8 mM Ca^++^ and 37°C. The experiments reported in Fig. 5 contain indications that the toxic conformation of hPrP90–231 is mainly due to small oligomers. Higher toxicity level and membrane conductance occur early, after 5, 15 and 24 hours and declines later, presumably due to the formation of bigger aggregates. Thus, it is realistic to think that high Ca^++^ level and/or high temperature work as catalyst to speed up *in vitro* a spontaneous reaction. In a healthy system the rate of spontaneous toxic PrP species formation is compatible with the ability to efficiently eliminate the misfolded proteins. It is also possible that the slow production of reactive peptides would favor the direct aggregation of monomeric PrP in amorphous big aggregates without the formation of harmful intermediated oligomeric form [64]. However, it should be also considered a different scenario. If the rate of PrP misfolded proteins formation drastically increases, all the system devoted to annihilation of aberrant proteins would be plugged and this will favored a harmful unbalanced situation. Misfolded proteins will increase in concentration allowing the formation of reactive oligomers before the peptides would reach the amorphous state of big aggregates. Small oligomers in their short lifespan will be able to react with the biological membrane causing unregulated ionic flows, most of the time resulting in a depolarization of the cell membrane potential. Depolarized neuronal cells could be considered a first stage, even if reversible, of the disease progression. In addition to that, the formation of membrane discontinuity could also be the pathway allowing extracellularly accumulated PrP peptides to leak in the cell to form aggregates in the cytosol. Cytotoxicity could be the result of both these factors. Pathogenic PrP, by changing the membrane voltage, is able to increase intracellular Ca^++^ concentration through the activation of both ion channels and ionotropic receptors. Thus, it is likely that elevation of intracellular Ca^++^ may interfere with internalized and neosynthesized PrP molecules causing a structural alteration within the cell. In this model we have the starting of a vicious circle in which altered PrP molecules affect intracellular membrane permeability to Ca^++^ that reaching high intracellular levels contribute to the generation of new toxic species that altogether with the altered Ca^++^ homeostasis induce synapse dysfunction and neuronal death.

In conclusion, we suggest that increased membrane permeability due to the structural rearrangement of PrP peptide is a spontaneous long lasting reaction. Increase of membrane conductance could be linked to pathological conditions once there is an overproduction of PrP misfolded protein. In this case the system is not capable to eliminate the dangerous peptide that, both for environmental conditions and for its concentration, is allowed to form highly reactive oligomers able to strongly interact with the cell membrane. The possibility to form high conductance ionic pathways could be also the vehicle to allow PrP protein inside the cell. Here the peptide could aggregate and form deposits [18]. Alternatively misfolded PrP could form oligomers and cause damage to internal membrane such as mitochondria, lysosomes and nuclear envelope, determine a slowly but inexorable collapse of the cell organization.

## Materials and Methods

Ethics Statement: N/A.

### Prion Samples

The protease-resistant core (PrP27–30) of PrPTSE extracted from brains of TSE-affected hamsters PrP27–30 was kindly provided by the Pocchiari’s group [31]. Recombinant prion peptide PrP 90–231 (hPrP90–231) was synthesized in *E. coli* and purified as previously reported [15]. The same procedure was used to purify the E200K and D202N hPrP90–231 mutants [17]. GST was purified from the empty pGEX-4T-2 vector following the same procedure used to purify hPrP90–231 [7].

### Cell Cultures and Treatments

SH-SY5Y human neuroblastoma cells (ICLC-Biological Bank and Cell Factory, IRCCS IST Genova, code HTL95013) were cultured in MEM/F12 (Euroclone, Milano, Italy) supplemented with 15% fetal bovine serum (Gibco-Invitrogen, Milano, Italy), 2 mM glutamine (Euroclone, Milano, Italy), pen/strep 100 mg/ml (Euroclone) and grown in 5% CO_2_ atmosphere at 37°C. Twenty-four hours after plating, unless otherwise stated, culture medium was replaced with fresh medium containing a reduced (2%) FBS content, to induce growth arrest and minimize spontaneous apoptosis that serum withdrawal causes in this cell model [32]. Cells were treated for 2 days with a single administration of hPrP90–231 directly to the culture medium to mimic the interaction of PrP^Sc^ deposits with the neurons as occurred *in vivo* [33].

### Survival Assay

Mitochondrial function, as index of cell viability, was evaluated by measuring the reduction of 3-(4,5-dimethylthiazol-2-yl)-2,5-diphenyltetrazolium bromide (MTT) (Sigma-Aldrich, Milano, Italy). The cleavage of MTT to a purple formazan byproduct by mitochondrial dehydrogenases was spectrophotometrically quantified, as reported [34]. Briefly, cells were incubated for 1 h with 0.25 mg/mL MTT in serum-free Dulbecco’s modified Eagle’s medium at 37°C; medium, was removed and formazan crystals dissolved in dimethyl-sulfoxide (DMSO). Absorbance values were measured at wave length of 570 nm. Experiments were performed in quadruplicate and repeated at least three times. Data are reported as mean values ± standard error (S.E.) Statistical analysis was performed by means of one-way ANOVA followed by Newman and Keuls test. A p value less than or equal to 0.05 was considered statistically significant.

### Electrophysiological Assay

Single-channel recordings from lipid bilayer were obtained using the Tip-Dip method. Patch clamp pipettes (Garner Glass 7052) were made using a P97 Sutter Instruments puller (Novato, CA), coated with Sylgard (Dow Corning, Midland, MI) and fire-polished to a tip diameter of 1–1.5 µm and 5–7 MOhm resistance. 1,2-diphytanoyl-sn-glycero-3-phosphocholine (Diph-PC, Avanti Polar Lipids, Inc., Birmingham, AL) was used to arise the lipid monolayer. An Axopatch 200 B amplifier and pClamp 8 (Molecular Device, Novato, CA) were used to record and analyze, digitized at 5 kHz and filtered at 1000 Hz the detected current. Membrane voltages (Vm) and ion currents were expressed considering relative to the pipette solution. An inward current is defined as a cation moving from the electrode to the bath chamber. Data are presented as mean ± SEM. Values obtained from different experiments were tested for statistical differences using independent two population t test or ANOVA routine (Origin software; Origin Lab, Northampton, MA). Different buffers were employed in the two compartments. The ion compositions of the *trans* solutions were defined in order to resemble the physiological composition (144 mM NaCl, 1.8 mM CaCl_2_, 1.2 mM MgCl_2_, 10 mM Hepes, pH 7.0), and the *cis* solution to mimic the cytoplasmic ion milieu (144 mM KCl, 0.1 mM CaCl_2_, 1.2 mM MgCl_2_, 1.1 EGTA, 10 mM Hepes, pH 6.2). Samples of hPrP90–231, *wt* and its E200K and D202N mutated forms employed in all Tip-Dip assays were made up to a final concentration of approximately 80 µM in a solution containing: 144 mM NaCl, 1.8 mM CaCl_2_, 1.2 mM MgCl_2_, 10 mM Hepes, pH 5.0 and DMSO 55%. 20 microliters of the stock solutions were added to 1 ml of the test solutions to a final concentration of 160 nM. In ion current measurements achieved in essaying the effect of Ca^++^, increasing amounts of CaCl_2_ (5, 10, 20, 50 mM) were added in the *trans* solution.

### Circular Dichroism (CD)

Spectra were measured on a Jasco J-600 spectropolarimeter, calibrated with camphorsulphonic acid [35]. Samples were diluted in 10 mM phosphate buffer, pH 7.2. Spectra were recorded between 200 and 250 nm with a 1 nm spectral step size, 0.5 nm bandwidth and 100 nm/min scan rate, using 1 mm quartz cell. Spectra represent the average of 10 scans subtracted of blank values (10 scans of buffer alone). The experiments were independently repeated three times.

### Thioflavin T (Th T) Binding Assay

Peptides was incubated with 10 µM Th T (Sigma-Aldrich) (in 20 mM NaOPO4, 0.15 M NaCl, pH 7.0) and fluorescence was monitored using Lambda Bio 10 spectrofluorometer (Perkin Elmer). Th T fluorescence was measured with excitation at 450 nm and emission from 460 to 560 nm with excitation and emission slits of 10 nm, as reported [36]. Spectra are reported subtracted of respective blank values.

### 2-(p-Toluidinyl)naphthalene-6-sulfonate (TNS) Binding Assay

This assay was performed with a Spex spectrofluorimeter (model Floromax) equipped with a temperature-controlled cell compartment maintained at 18°C using the hydrophobicity fluorescent probe TNS, as reported [37]. In particular, the fluorescence emission of the complex hPrP90–231-TNS (1 µM of peptide plus 100 µM of TNS, dissolved in 2 mM phosphate buffer pH 6.0) was recorded between 400 and 510 nm, with an excitation wavelength at 365 nm. The spectra were reported subtracted of the respective blank solution containing 100 µM TNS plus 1.8, 5, or 10 mM Ca^++^.

### Intrinsic Fluorescence Measurements

Fluorescence spectra were measured with a Lambda Bio 10 spectrofluorimeter (Perkin-Elmer). Tryptophan emission from of hPrP90–231 was recorded between 295 and 420 nm, with excitation at 295 nm. The spectra were reported subtracted of the respective blank values [37].

### hPrP90–231 Proteinase K (PK) Resistance Assay

hPrP90–231 resistance to proteolysis was revealed by immunoblotting [7]. Digestion was performed treating 100 µg proteins with increasing concentrations of PK (1, 2, 10 µg/ml) for 30 minutes at 37°C. Digestion was stopped by boiling samples in Laemmli buffer. Digestion profile was detected subjecting samples to 15% SDS-PAGE followed by immunoblotting using the anti PrP mouse monoclonal antibody 3F4 (epitope corresponding to amino acids 109–112 in the human PrP: Signet Lab, London, UK).

### Computational Details: Structural Analyses of PrP^C^ Wild Type and E200K Mutant

The experimental NMR structures of the recombinant wild type hPrP90–230 and the hPrP90–231 E200K mutant were taken from the PDB archives, entry code 1QM0 [38] and 1FKC [39], respectively. The selected entries were graphically inspected and analyzed to find possible structural mismatches by using the molecular modelling software Maestro [40].

A geometry minimization has been preliminarily performed for both proteins, starting from the experimental structures, by using MacroModel [41] with the OPLS-AA force field [42], [43] and a gradient threshold of 0.05 kJ mol^−1^ Å^−1^. The water environment was simulated with the GB/SA implicit solvation model implemented in the program [44].

The structures so obtained correspond to local minima of the potential energy surfaces (PES) for the two proteins and were then employed as input for large-scale low mode (LLMOD) searches [45], [46] allowing the characterization of further local minima in a wider section of their PES. The LLMOD calculations were carried out by imposing the default set-up to the search variables, carrying out 1000 Monte Carlo steps before stopping, setting 3.0 and 6.0 Å as minimum and maximum distance step, respectively, and imposing a convergence limit to the local minimization steps of 1.0 kJ mol^−1^ Å^−1^, to increase the time performances of the search.

The exploration of the first 30 pure normal modes corresponding to the eigenvectors of the input structure Hessian with the lowest frequencies was carried out by the global search option, allowing each Monte Carlo step to begin with the preceding structure, provided its energy is within 100 kJ mol^–1^ of the global minimum.

The LLMOD outputs were subsequently minimized and characterized as unique structures within a gradient threshold of 0.050 kJ mol^−1^ Å^−1^, using the multiple minimization module implemented in MacroModel. The multiple minimization outputs were further filtered to eliminate high energy conformation and, eventually, the structure corresponding to the global minimum was taken as representative of the water solution structure for each PrP^C^ form, and hereafter referred to as models I and II.

These two models were then superimposed by the minimization of root mean square deviations (RMSD) of Cα backbone positions. The resulting structures were centered and aligned with respect to center of mass and moment of inertia, using the dedicated tool of Maestro. The direction of the smallest principal moment of inertia, used as x-axis in the workspace coordinates of Maestro, is almost aligned with H3, the longest among the three helices characterizing the three-dimensional structure of PrP^C^.

Preliminarily, the calculated structures of models I and II were graphically inspected with Maestro to retrieve qualitative information on the main differences and similarities between the two PrP^C^ forms. In particular, we focused on the distribution of charged residues on the whole protein surface because of their major contribution to the molecular electrostatic potential (MEP). We also characterized salt bridges and most stable hydrogen bonds (involving charged residues) in terms of intra or interdomain interactions which are expected to play a major role in the stabilization of the tertiary structure. A more detailed analysis was then performed by comparing the MEPs of models I and II in the space surrounding the superimposed protein models (I U II) by means of a in-home Perl script (Fig. S1). Both MEPs were probed on the same three-dimensional grid around the (I U II) superimposed structure and characterized by a grain of 0.35 Å and dimensions of 66×47×40 Å, large enough to ensure an adequate mapping of MEP in the space surrounding the entire protein surface. The electrostatic potential on a grid point *r* was calculated by using the classical expression:
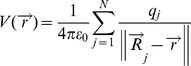
where *N* is the number of protein atoms, and 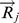 and *q_j_* the vector associated to position of *j* ^th^ protein atom, and its OPLS-AA charge, respectively [44].

The MEP similarity between model I and II was then evaluated at different portion of the grid by the calculation of Carbó similarity index (σ*_C_*) [47] using the following formula:
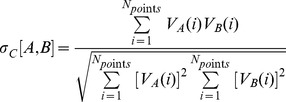
where *V*_I_(*i*) and *V*_II_(*i*) are the corresponding MEPs calculated at *i* ^th^ point, and *N_points_* is the number of grid points considered for the calculation of similarity.

Moreover, because the important role played by the H3 helix, characterizing the whole protein frame, we performed our similarity analysis with respect to its axis (approximately taken as the x-axis in the workspace coordinates of Maestro). Accordingly, the grid space was partitioned into slices in the yz plane, i.e. perpendicular to the x axis, and σ*_C_* was calculated on each slice (defined as a set of 6 layers of points sharing the same x coordinate) by including only the points external to the protein surface. The whole grid space was partitioned into 34 slices and the whole procedure was parallelized thus providing higher computational performance. An estimate of *σ*_C_ as a function of the position of the slice along the x axis was thus obtained including in the previous formula only the points of single yz-slices.

### Ca^2+^ Binding to Wild Type PrP^C^

The building of a molecular model for the binding of the Ca^++^ to the PrP^C^ wild type protein (model III) was carried out on the basis of an inspection of the MEP and of the distribution of potentially coordinating side chains around the *w.t.* protein surface, suggesting a plausible metal binding site in the close proximity of Arg 156, His187, Thr191 and Glu196 (see Results and Discussion).

Model I (see above) was used as a template to generate an initial guess of model III, by using the Maestro graphical interface. Ca^++^ binding site was generated by moving the side chain of Arg 156 toward the external bulk, using the “quick torsion” tool of Maestro. The Ca^++^ ion was then inserted in the site left by the Arg156 side chain and suitably placed to bind the imidazolic nitrogen of His 187, the phenolic oxygen of Thr 191, and the backbone carbonyl oxygen of Arg 156 itself. This structure underwent the same computational treatment of models I and II (see above) consisting of local minimization, LLMOD search, and multiple minimization, which eventually led to model III.

Model III was then compared with models I and II with the same procedure employed for the comparison between models I and II. After superposition to model I or II, model III underwent graphical inspection and the mapping of MEP by using the same approach employed in the I/II comparison. Carbó similarity index was calculated for both I/III and II/III comparisons as a function of the position along the x axis and the results were compared to the corresponding I/II analyses.

## Supporting Information

Figure S1 **Scheme of the Perl script algorithm for the mapping of MEP at the protein surface according to the partitioning scheme into slices along the x axis.**
(PDF)

Figure S2 **Ribbon view of protein model I (dark green), II (light cyan) and III (orange) after superposition through minimization of Cα RMSD.** The grid points (red spheres) corresponding to the two minima of I/II similarity, the hypothesized Ca^++^ binding site and the mutation site are also displayed.
(PDF)
