# Supplementary figures and images for: Calcium Binding Promotes Prion Protein Fragment 90–231 Conformational Change toward a Membrane Destabilizing and Cytotoxic Structure
Source: PLoS One. 2012 Jul 11;7(7):e38314. doi: 10.1371/journal.pone.0038314 (PMC3394757; doi:10.1371/journal.pone.0038314)

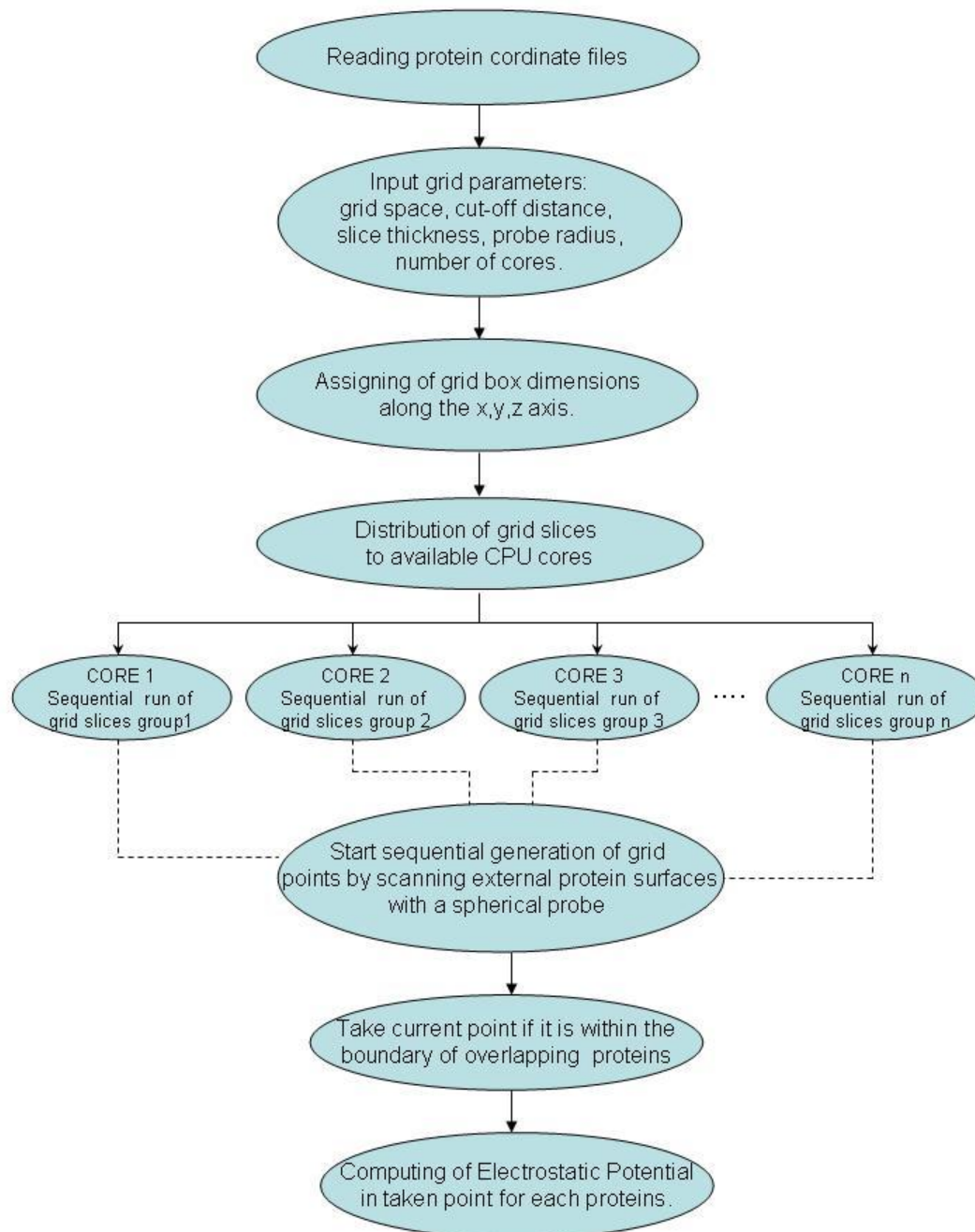

Supplement: Figure S1 — Scheme of the Perl script algorithm for the mapping of MEP at the protein surface according to the partitioning scheme into slices along the x axis. (PDF) [file pone.0038314.s001.pdf]
