# Supplementary material for: Calcium Binding Promotes Prion Protein Fragment 90–231 Conformational Change toward a Membrane Destabilizing and Cytotoxic Structure
Source: PLoS One. 2012 Jul 11;7(7):e38314. doi: 10.1371/journal.pone.0038314 (PMC3394757; doi:10.1371/journal.pone.0038314)
Supplement: Figure S2 — Ribbon view of protein model I (dark green), II (light cyan) and III (orange) after superposition through minimization of Cα RMSD. The grid points (red spheres) corresponding to the two minima of I/II similarity, the hypothesized Ca++ binding site and the mutation site are also displayed. (PDF) [file pone.0038314.s002.pdf]

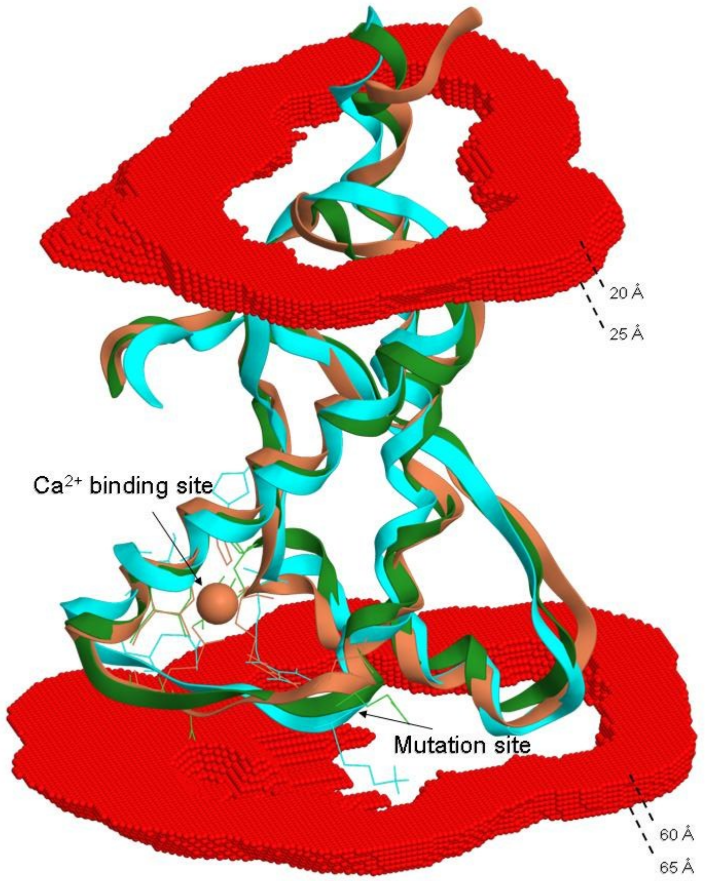

Figure S2 – Ribbon view of protein model I (dark green), II (light cyan) and III (orange) after superposition through minimization of Cα RMSD. The grid points (red spheres) corresponding to the two minima of I/II similarity, the hypothesized Ca^{2+} binding site and the mutation site are also displayed.
